# Biomarkers in subtypes of mild cognitive impairment and subjective cognitive decline

**DOI:** 10.1002/brb3.776

**Published:** 2017-07-28

**Authors:** Carl F. Eliassen, Ivar Reinvang, Per Selnes, Ramune Grambaite, Tormod Fladby, Erik Hessen

**Affiliations:** ^1^ Department of Psychology University of Oslo Oslo Norway; ^2^ Department of Neurology Akershus University Hospital Lørenskog Norway; ^3^ Institute of Clinical Medicine Campus Ahus University of Oslo Oslo Norway

**Keywords:** Aβ42, biomarkers, cortical thickness, CSF, metabolism, mild cognitive impairment, MRI, neuropsychology, positron‐emission tomography (PET), subjective cognitive decline, tau

## Abstract

**Objectives:**

Preclinical Alzheimers disease (AD) patients may or may not show cognitive impairment on testing. AD biomarkers are central to the identification of those at low, intermediate, or high risk of later dementia due to AD. We investigated biomarker distribution in those identified as subjective cognitive decline (SCD), amnestic (aMCI), and nonamnestic (naMCI) mild cognitive impairment (MCI) subtypes. In addition, the clinical groups were compared with controls on downstream neuroimaging markers.

**Materials and Methods:**

Cerebrospinal fluid (CSF) amyloid‐β42 (A β42) and total tau (t‐tau), phosphorylated tau (p‐tau), fluorodeoxyglucose (FDG), positron‐emission tomography (PET), and MRI neuroimaging measures were collected from 116 memory clinic patients. They were characterized as SCD, aMCI, and naMCI according to comprehensive neuropsychological criteria. ANOVAs were used to assess differences when biomarkers were treated as continuous variables and chi square analyses were used to assess group differences in distribution of biomarkers.

**Results:**

We did not find any between group differences in Aβ42, nor in p‐tau, but we observed elevated t‐tau in aMCI and SCD relative to the naMCI group. Significantly lower cortical glucose metabolism (as measured by FDG PET) was found in aMCI relative to SCD and controls, and there was a trend for lower metabolism in naMCI. Significant thinner entorhinal cortex (ERC) was found in aMCI and SCD. As expected biomarkers were significantly more frequently pathological in aMCI than in naMCI and SCD, whereas the naMCI and SCD groups displayed similar pathological biomarker burden.

**Conclusions:**

aMCI cases show the most pathologic biomarker burden. Interestingly naMCI and SCD subjects show similar levels of pathological biomarkers albeit the former displayed neuropsychological deficits. That the latter group may represent a risk group is supported by our observation of both elevated CSF tau and thinner ERC relative to controls.

## 1 INTRODUCTION

Subjective cognitive decline (SCD) entails a transient period of subjectively perceived subtle cognitive deficits, but with normal performance on neuropsychological testing. SCD is proposed to precede mild cognitive impairment (MCI) where accumulated neuropathology contributes to mildly reduced neuropsychological test results. Both conditions are recognized as tentative Alzheimers disease (AD) predementia stages (Albert et al., 2011; Jessen et al., 2014). Pathological cerebrospinal fluid (CSF) biomarkers, cortical glucose hypometabolism, and atrophy of specific brain regions reflect pathophysiological processes taking place years before manifest AD (Sperling et al., 2011). Biomarkers are differentially linked with clinical progression (Caselli & Reiman, 2013; Dubois et al., 2014; Selnes et al., 2013), and how AD biomarkers distribute across clinical subtypes may reveal pathological underpinnings of subjective and objective cognitive symptoms and deficits.

The National Institute on Ageing and Alzheimer's disease Association (NIA‐AA) has put forward diagnostic research criteria for incipient AD (Albert et al., 2011). The criteria encompass cognitive impairment with additional biomarkers of reduced CSF amyloid beta (Aβ42) or increased cortical Aβ plaques, increased CSF tau levels, temporoparietal [^18^F]‐fluorodeoxyglucose‐positron‐emission tomography (FDG‐PET) hypometabolism, and atrophy to medial temporal structures measured with MRI, as criteria contributing to a MCI‐AD diagnosis. Depending on biomarker profiles MCI due to AD may qualify into different risk groups: low AD likelihood group (no biomarker pathology), high AD likelihood group (Aβ42 pathology and a biomarker of neurodegeneration), intermediate, or suspected non‐AD pathology (SNAP). In the latter group at least one pathologic marker of neurodegeneration is required, but no Aβ42 pathology, which is theorized to occur prior to neurodegeneration in classical AD development (C. R. Jack et al., 2013). The other major consensus research criteria were formulated by the International Working Group (IWG) (Dubois et al., 2007), and later refined two times to improve the diagnostic framework in terms providing criteria for different phenotypes such as typical and atypical AD (Dubois et al., 2010, 2014). Although the NIA‐AA and IWG diagnostic algorithms emphasize biomarkers, they differ in terms of nomenclature, staging, and the interpretation of biomarker findings (Jack et al., 2016; Vos et al., 2015). Recently an unbiased descriptive classification system which is agnostic to disease mechanisms has been proposed to account for accumulating evidence of increased AD risk irrespective of which pathological biomarker type that is present (Jack et al., 2016).

However, MCI subjects identified as SNAP (NIA‐AA definition) have been postulated to possibly represent those at risk of developing dementia phenotypes other than AD, such as for example frontotemporal dementia, vascular dementia, or dementia with lewy bodies (Caroli et al., 2015). Diagnostic groups are formed according to the proposed model where AD pathogenesis develops as sequential cascade of events initiated by Aβ42 pathology, followed by tau pathology and subsequent cortical glucose hypometabolism and neurodegeneration (Jack et al., 2013).

Although the NIA‐AA requires objective cognitive test impairment within any domain (Albert et al., 2011), subtyping MCI according to which cognitive deficits are presented have been postulated to aid the identification of MCI due to incipient AD from other types of dementia (Petersen et al., 2014). MCI with amnestic symptoms (aMCI) have traditionally been linked with later AD, whereas those where nonamnestic deficits predominate (naMCI) have been suggested to better represent SNAP (Petersen et al., 2014; Vos et al., 2013). One study reported that for aMCI progression to AD was 38% relative to 20% for those identified as naMCI, whereas progression to non‐AD dementias was similar for both groups (Vos et al., 2013). In a later multicenter study it was found similar AD predictive accuracy between aMCI and naMCI when both groups displayed pathological biomarkers, and a high conversion to AD of approximately 20% in those identified as SNAP (Vos et al., 2015).

Neuropsychological testing is central to MCI classification, but different characterization schemes have been utilized**.** The traditional approach for neuropsychological MCI classification requires one impaired test measure, but has been shown susceptible to false‐positive diagnostic errors. (Bondi & Smith, 2014; Edmonds et al., 2014a). This may be due to the statistical likelihood of obtaining at least one reduced score when several tests are administered (Binder, Iverson, & Brooks, 2009) or the effects of psychological factors such as anxiety and depression (Comijs, Deeg, Dik, Twisk, & Jonker, 2002). However, when MCI classification requires impairment on two neuropsychological tests in the same cognitive domain, this has been shown to substantially reduce the number of false‐positives (Bondi et al., 2014; Clark et al., 2013; Edmonds et al., 2014a).

We investigated the distribution of AD biomarkers in a young clinical sample of comprehensively defined aMCI, naMCI, and SCD subjects. Biomarkers included CSF Aβ42, total tau (t‐tau), phosphorylated tau (p‐tau), FDG‐PET cortical metabolism, and entorhinal cortex (ERC) thickness as measured with MRI. We set to explore pathological biomarker distribution between aMCI, naMCI, and SCD. Because MCI defined according to a comprehensive neuropsychological classification scheme has been shown to reduce the risk of false‐positive diagnostic errors and display similar prevalence of CSF biomarker pathology irrespective of which cognitive impairments predominate (Bondi et al., 2014), we expected aMCI and naMCI to show a higher biomarker burden than SCD patients. We were particularly interested in whether naMCI displayed a more nonamyloid biomarker profile relative to aMCI, and thus more representative of those commonly referred to as SNAP.

## 2 MATERIAL AND METHODS

### 2.1 Research participants

Participants were referred to our memory clinic by their general practitioner or by self‐referrals. Inclusion criteria were subjective cognitive complaints for minimum 6 months, preserved general function, no or very mild deficits in activities of daily living (ADL), Global Deterioration Scale (GDS) score of 2 or 3 (Reisberg, Ferris, de Leon, & Crook, 1982), and Clinical Dementia Rating (CDR) scores of maximum 0.5 (Morris, 1993). Mini Mental Status Examination (MMSE) score of ≥25 (Folstein, Folstein, & McHugh, 1975). Exclusion criteria included clinical depression, neurological disease, previous brain injury, cardiovascular events, and substance abuse. In total 78 subjects were classified as MCI. Thirty‐eight patients without objective cognitive impairment were characterized as SCD. One patient with MMSE score below 25, four patients with frontotemporal dementia, and six patients with vascular pathology evident from MRI were excluded. Spouses or partners of participating patients were included as controls provided a GDS score of 1 and neuropsychological performance within the normal range. The project was approved by the South‐Eastern Norway committee for medical research ethics. Participants’ consent was obtained according to the Declaration of Helsinki (Declaration of Helsinki, 1991).

### 2.2 Neuropsychological classification

Neuropsychological MCI classification was conducted in accordance with the “comprehensive criteria” (Bondi et al., 2014; Jak et al., 2009), which require two test scores within the same cognitive domain to be ≤1 standard deviation (*SD*) below published normative data. Neuropsychological tests of memory, visuoconstruction, attention/processing speed and executive functioning were administered. Tests included Rey Auditory Verbal Learning Test, Wechsler Logical Memory Test Revised (Wechsler, 1997), Rey Complex Figure test (copy and delayed recall), Letter number sequencing test (WAIS III) (Wechsler, 1997), Trail Making Test A, Trail Making Test B, COWAT phonetic fluency test, and Color Word Interference Test (D‐KEFS). Fifty‐three patients were classified as aMCI and 26 patients were classified as naMCI. Remaining 38 patients who did not meet our neuropsychological diagnostic criteria, but fulfilled clinical screening were categorized as SCD.

### 2.3 CSF biomarkers

CSF concentrations of Aβ42 and t‐tau were quantified with ELISAs; Innotest® Β‐amyloid 1‐42 Innotest® hTau Ag (Vanderstichele et al., 2000), and Innotest® phosphoTau (181P) (Vanmechelen et al., 2000) (Fujirebio Europe, Gent, Belgium). Analyses were carried out in accordance with manufacturer instructions at the national reference laboratory for these tests at the Department of InterdiSCDplinary Laboratory Medicine and Medical Biochemistry, Akershus University Hospital. Lumbar puncture for CSF collection was mostly performed between 8 a.m. and 12 a.m, at the L3/L4, L4/L5, or L5/S1 interspace. Samples were analyzed individually. The first 4 ml CSF was used for routine clinical investigations, whereas the next 1.5 ml CSF was collected in polypropylene tubes and centrifuged at 2,000 *g* for 10 min within 4 hr after collection. The 1.5 ml CSF was stored at −80°C prior to analysis of the CSF biomarkers Aβ42, t‐tau, and p‐tau. CSF Aβ42 values were considered pathological if <700 ng/L. This cutoff was validated in against Flutemeramol‐PET in an extension of the same cohort (Almdahl et al., 2017). CSF total tau values were considered abnormal if T‐tau exceeded 300 ng/L for patients less than 50 years of age, more than 450 ng/L for patients from 50 to 69 years and above 500 ng/L for patients over 70 years (Sjogren et al., 2001), p‐tau was considered abnormal if values exceeded 80 ng/L.

### 2.4 FDG‐PET scanning and analyses

[^18^F]‐fluorodeoxyglucose‐PET imaging was obtained with a Biograph 16 PET/CT scanner (Siemens). All participants fasted for at least 4 hr prior to scanning, and displayed blood glucose in the range 4.3–6.8 mmol/L. An intravenous bolus of 200 MBq 18F‐FDG was injected and subjects rested for 45 min before scanning. A low‐dose nondiagnostic CT scan was performed followed by PET scanning. PET acquisition was performed in 3D mode with only single axial position for 15 min. Attenuation and scatter corrections were performed. The images were reconstructed by an iterative technique (5 iterations, 8 subsets) using a Gaussian smoothing filter with full width at half maximum (FWHM) of 3.5 mm. The image format was 256 × 256. FDG‐PET frames were registered to the corresponding MRI volume. PET signals were averaged within each region of interest (ROI) based on structural MRI parcellations (Desikan et al., 2006; Fischl, 2004) and normalized to activity in the brainstem. The ROIs included the inferior temporal, inferior parietal and posterior cingulate, regions previously found sensitive to early AD‐related hypometabolism (Landau et al., 2011). FDG‐PET signal was considered hypometabolic if it fell 1 *SD* below the average age and gender adjusted mean value.

### 2.5 Cortical thickness MRI segmentation of entorhinal cortex

MRI scans were obtained from two different scanners. The first scanner was a Siemens Symphony 1.5T. T1‐weighted volumetric MP‐RAGE sequence was collected (TR/TE/TI/FA = 2,730/3.19/1,100/15◦, matrix = 256 × 192), consisting of 128 sagittal slices, thickness  = 1.33 mm, in‐plane resolution of 1.0 × 1.33 mm. The second scanner was a Siemens Espree 1.5 T. One 3D MP‐RAGE, T1‐weighted sequence was collected (TR/TE/TI/FA = 2,400/3.65/1,000/8°, matrix = 240 × 192), consisting of 160 sagittal slices, thickness = 1.2 mm, in‐plane resolution of 1 × 1.2 mm. Cortical reconstruction and volumetric segmentation were performed with the FreeSurfer image analysis suite version 5.3 (http://surfer.nmr.mgh.harvard.edu/). This includes segmentation of the deep gray matter volumetric structures (Fischl et al., 2009) and parcellation of the cortical surface (Desikan et al., 2006; Fischl, 2004). For this study only ROI thickness values of the ERC were calculated using methods based on ultrahigh resolution *ex vivo* applied to *in vivo* MRI (Fischl et al., 2009). Four individuals were scanned on both scanners to investigate potential bias. Mean differences in cortical thickness were generally within ±0.1 mm across the brain surface. Intraclass correlation coefficients (ICC) for ERC thickness between the two scanners were ~1.

### 2.6 Statistical analyses

The Statistical Package for Social Sciences (SPSS 21.0) was used. Between‐group characteristics were compared with analysis of variance (ANOVA) for continuous variables, Bonferroni corrected post hoc tests for between group comparisons of continuous biomarker data, Kruskal–Wallis test for MMSE, and Chi‐square tests in case of dichotomous biomarker variables.

## 3 RESULTS

ANOVA showed no significant between group differences between aMCI, naMCI, and SCD with respect to age, education and MMSE scores (Table 1). There was no significant between group difference in education and MMSE.

**Table 1:** Demographics and MMSE for aMCI, naMCI, SCD, and controls

	aMCI (*n* = 54)	naMCI (*n* = 27)	SCD (*n* = 38)
Age (*SD*)	61.9 (7.8)	60.7 (7.8)	59 (8.3)
Education (*SD*)	12.3 (2.9)	12.7 (3)	12.7 (3.1)
MMSE (*SD*)	27.9 (1.4)	28.2 (1.5)	28.2 (1.7)

MMSE, Mini‐Mental Status examination; SCD, subjective cognitive decline.

ANOVA was performed to investigate between group differences in aMCI, naMCI, and SCD. Continuous biomarker data were adjusted for effects of gender and age. Bonferroni corrected post hoc analyses were applied to subtype comparisons. There were no significant between group differences in Aβ42 *F*(2, 110) = 0.566, *p* = .57. Significant ANOVA between group differences were found for total tau *F*(2, 90) = 4.06, *p* = .021. Significant higher total tau was found in aMCI (mean = 419.6, *SD* = 253.4) relative to naMCI (mean = 268.2, *SD* = 126.8), *p* = .041, and between, SCD (391.8, *SD* = 324) and naMCI (mean = 268.2, *SD* = 126.8), *p* = .046. While between group differences in p‐tau were not significant *F*(2, 110) = 2.6, *p* = .079. Significant between group differences was found for FDG‐PET *F*(3, 163) = 8.94, *p* = .000. aMCI (−0.48, *SD* = 1.09) showed significant lower standardized age adjusted FDG‐PET values than SCD (0.42, *SD* = 0.89), *p* = .000, and controls (0.32, *SD* = 0.75) *p* = .000. Significant between group differences was found for MRI cortical thickness of the ERC *F*(2, 163) = 5.88, *p* = .001. aMCI (−0.26, *SD* = 1.02) showed significant thinner ERC than controls (0.48, *SD* = 0.188) *p* = .001, and SCD (−0.15, *SD* = 0.87) than controls, *p* = .016.

Chi square tests of independence were performed between aMCI, naMCI, and SCD for the selected biomarkers. There was no significant differences in pathological Aβ42 frequency between aMCI, naMCI, and SCD χ^2^ (2, *N *=* *110) = 3.1, *p *=* *.21. There were no significant between group differences in t‐tau χ^2^ (2, *N *=* *110) = 3.47, *p *=* *.18, but a significant group difference was found for p‐tau χ^2^ (2, *N *=* *110) = 8.95, *p *=* *.01. We reran the analyses and found that p‐tau pathology was significantly more frequent in aMCI than naMCI χ^2^ (1, *N *=* *77) = 7.29, *p *=* *.007, and significantly more frequent in aMCI than in SCD χ^2^ (1, *N *=* *86) = 4, *p *=* *.046. Significant between group differences were found for pathological FDG‐PET frequency χ^2^ (2, *N *=* *110) = 8.79, *p *=* *.012. When we reran the analyses we did not find a significant difference between aMCI and naMCI, but FDG‐PET under cutoff values were significantly more frequent in aMCI than in SCD were χ^2^ (1, *N *=* *82) = 8.7, *p *=* *.003, but not in naMCI compared to SCD χ^2^ (1, *N *=* *57) = 3.24, *p *=* *.072 was significantly more frequent in MCI (30.7%) than in SCD (9.2%), χ^2^ (2, *N *=* *108) = 5.84, *p *<* *.05. We found no between group differences with respect to ERC thickness χ^2^ (2, *N *=* *110) = 1.73, *p *=* *.422.

Chi square tests of independence for biomarker counts (0–4 possible biomarkers) was not significant χ^2^ (2, *N *=* *116) = 14.517, *p *=* *.069. However, there was a significant (8.691, *p *=* *.003) linear‐by‐linear association showing that there is a significant association between diagnostic group and number of biomarkers (Table 2).

**Table 2:** Proportion of patients in MCI and subjective cognitive decline (SCD) with 0–4 pathologic biomarkers

	aMCI (*n* = 53)	naMCI (*n* = 26)	SCD (*n* = 38)
0	26.4%	40.7%	41.2%
1	26.4%	33.3%	35.3%
2	18.9%	22.2%	20.6%
3	24.5%	3.7%	2.9%
4	3.8%	0%	0%

Percentage of clinical groups with 0–4 pathological biomarkers.

Chi square test between diagnostic groups and percentage of patients with less than two pathological biomarkers and those with two or more pathological biomarkers. We found that having two or more pathological biomarkers were significantly more common in the aMCI group χ^2^ (2, *N *=* *116) = 6.386, *p *=* *.041, Cramer's V = 0.237 than the naMCI and SCD groups (Table 3).

**Table 3:** Percentage of clinical groups with <2 and >2 pathological biomarkers

	<2 Biomarkers	≥2 Biomarkers
aMCI	52.8%	47.2%
naMCI	74.1%	25.9%
SCD	76.5%	23.5%

SCD, subjective cognitive decline.

## 4 DISCUSSION

In our relatively young clinical sample of aMCI, naMCI, and SCD subjects we observed somewhat different AD biomarker profiles based on comparison according to levels or dichotomization according to cutoff values. We found that t‐tau was significantly elevated in aMCI and SCD as compared with naMCI. P‐tau levels were not significantly different across groups. Elevated t‐tau elevated in aMCI and SCD relatively to naMCI when the variables were analyzed continuously, has previously been observed in SCD in that progress clinically (Hessen et al., 2015), and was not unexpected. Although between group differences in p‐tau did not reach statistical significance we observed that there was a trend toward significance (*p* = .079). However, even though t‐tau and p‐tau correlate, they reflect different pathophysiological processes (Amlien & Fjell, 2014).

Among the clinical groups FDG‐PET metabolism were significantly lower in aMCI and naMCI than in SCD, whereas entorhinal cortical thickness was expectedly thinner in aMCI and SCD subjects relative to controls. That we found lower FDG‐PET metabolism in the MCI subgroups aligns with previous findings which indicates that that regional hypometabolism is closely linked with clinical progression (Ewers et al., 2014; Herholz, Boecker, Nemeth, & Dunn, 2013). On the other hand, we only found significant thinning of the ERC in aMCI and SCD. This structure is particularly involved with memory function and one of the earliest brain structures to degenerate in AD (Killiany et al., 2002).

A somewhat different pattern was seen with respect to proportion of patients who displayed pathological biomarkers according to dichotomized cutoff values. While Aβ42 were not significantly different between the groups, the pattern previously observed when all data were analyzed continuously was reversed for t‐tau and p‐tau. With dichotomized biomarker data we observed that pathologic t‐tau was not significantly different between groups, but pathologic p‐tau were significantly more frequent in aMCI than both naMCI and SCD. Pathologic FDG‐PET values were more frequently observed in aMCI than in SCD, whereas we did not find any significant differences with respect to frequency of ERC thickness between the groups.

Following a recently proposed alternative to the NIA‐AA AD risk factor staging procedure (Edmonds, Delano‐Wood, Galasko, Salmon, & Bondi, 2015), we compared aMCI, naMCI, and SCD with respect to the total number of pathologic biomarkers. There was a significant association for higher biomarker counts in the aMCI group, whereas the naMCI and SCD groups showed similar numbers of pathologic biomarkers across all stages (0–4 biomarkers). That the naMCI group did not display significantly higher number of pathologic biomarkers than SCD was unexpected since we used a neuropsychological classification scheme that has been shown less susceptible to include MCI subjects that revert back to normal cognitive functioning on follow‐up and less susceptible to false‐positive diagnostic errors. In contrast the SCD construct has been argued to represent a heterogeneous population partly due to the multitude of factors that contribute to perceived cognitive problems (Comijs et al., 2002; Hessen et al., 2017) and validity issues associated with subjective cognitive concerns (Edmonds et al., 2014b). On the contrary, individuals without objective cognitive impairment and cerebral amyloid pathology may experience heightened awareness of mild memory deficits (Vannini et al., 2017). Since we found similar biomarker counts in naMCI and SCD this lend support to the notion of SCD as a potential preclinical condition (Jessen et al., 2014). Lastly, we set a threshold to two or more biomarkers since this has been shown to be a critical biomarker threshold for those that progress clinically (Edmonds et al., 2015). We found that the aMCI group contained a significantly higher number of individuals with biomarker pathology than naMCI and SCD.

A substantial proportion of patients did not show biomarker pathology, even among the MCI subjects which were classified according to a stringent neuropsychological criterion. However, this aligns with others who have found large proportions of predementia patients without positive AD biomarkers (Alexopoulos et al., 2014). There may be several reasons for a high frequency of patients without biomarker pathology. First, cut‐off values dichotomizing continuous data may miss “near‐pathologic” cases. Second, the strength of association between specified biomarkers and cognitive impairment varies between individuals (Boyle et al., 2013). Lastly, other biomarkers than those established in AD research may have been more important. For example, we have previously found diffusion tensor imaging to surpass CSF markers as predictor of cognitive decline and atrophy at follow‐up (Selnes et al., 2013).

As we expected the aMCI group presented with a significantly higher burden of pathological biomarkers, but unexpectedly we found that the naMCI group was similar to the SCD group with respect to the number of pathological biomarkers. As evidence of objective cognitive impairment is required for MCI classification, SCD individuals may run the risk of clinical negligence. That SCD showed elevated total tau and cortical thinning of the ERC and similar biomarker counts as naMCI support the notion that subjectively reported cognitive decline may represent a preclinical AD condition (Jessen et al., 2014).

## CONFLICT OF INTEREST

The authors declare no financial or other conflict of interests.
